# Performance of the AUB-HAS2 cardiovascular risk index in coronary artery disease: a multicenter retrospective cohort study

**DOI:** 10.1080/07853890.2026.2664250

**Published:** 2026-04-24

**Authors:** Xiaolin Li, Congying Wang, Haodong Jiang, Jia Zhu, Runzhe Wu, Yongquan Niu, Feiyu Chen, Yunpeng Jin

**Affiliations:** ^a^ Department of Nutrition, The Fourth Affiliated Hospital of School of Medicine, and International School of Medicine, International Institutes of Medicine, Zhejiang University, Yiwu, China; ^b^ Department of Cardiology, The Fourth Affiliated Hospital of School of Medicine, and International School of Medicine, International Institutes of Medicine, Zhejiang University, Yiwu, China

**Keywords:** AUB-HAS2 cardiovascular risk index, coronary artery disease, revised cardiac risk index, preoperative cardiovascular evaluation, non-cardiac surgery

## Abstract

**Background:**

While the American University of Beirut (AUB)-HAS2 cardiovascular risk index has emerged as a novel tool for preoperative risk stratification, its performance specifically in coronary artery disease (CAD) patients warrants further validation. This study aimed to evaluate the performance of AUB-HAS2 index specifically in CAD patients.

**Methods:**

In this multicenter retrospective cohort study, we enrolled consecutive adult patients with documented CAD who underwent non-cardiac surgery between 2013 and 2024 at two tertiary academic medical centers in Zhejiang, China. The primary outcome was a composite of perioperative cardiovascular events (PCE), including all-cause death, myocardial infarction, or stroke, occurring intraoperatively or during postoperative hospitalization.

**Results:**

Among 10,294 participants, 374 (3.6%) experienced PCEs. The incidence of PCEs increased steadily with the increase in AUB-HAS2 index (0.3%, 2.3%, 5.2%, 10.1%, and 21.7% for AUB-HAS2 index of 0, 1, 2, 3, and > 3, respectively; p value for trend < 0.001). The AUB-HAS2 index showed significantly better discrimination than the revised cardiac risk index (RCRI) (C-statistic: 0.765 vs. 0.689; *p* < 0.001), with consistent performance across subgroups and better calibration. Decision curve analysis revealed enhanced clinical utility across clinically relevant thresholds.

**Conclusions:**

The AUB-HAS2 index demonstrates improved predictive performance compared to the RCRI in CAD patients, supporting its clinical adoption for preoperative cardiovascular risk stratification in this high-risk population.

## Background

Perioperative cardiovascular events (PCE)—including death, myocardial infarction, and stroke—are a leading cause of morbidity and mortality in patients with coronary artery disease (CAD) undergoing non-cardiac surgery [1]. Annually, more than 50 million such procedures are performed worldwide in patients with established CAD [2], with PCEs occurring in over 3% of cases—a risk more than double that of the general surgical population [3–7]. Given this elevated risk, preoperative cardiovascular assessment is critical for optimizing perioperative management in this high-risk cohort [8].

The revised cardiac risk index (RCRI) remains the most widely used risk stratification tool due to its simplicity and extensive validation [9]. However, it showed limited predictive performance for PCEs in Chinese patients over 65 years old with CAD [10].

Recently, the American University of Beirut (AUB)-HAS2 cardiovascular risk index has emerged as a promising alternative. Like the RCRI, it is simple to apply, but it demonstrates improved discriminatory power, enabling rapid and effective risk stratification (low, intermediate, or high) in patients undergoing non-cardiac surgery [11]. Although validated in general surgical cohorts [12], vascular surgery patients [13], and a prospective cohort [14], its performance specifically in CAD patients—a high-risk subgroup that would benefit most from accurate risk stratification—remains unknown.

To address this gap, we conducted a multicenter retrospective analysis to validate the AUB-HAS2 index specifically in CAD patients undergoing non-cardiac surgery, and compare its performance against the RCRI. This study aimed to provide evidence on the adoption of this novel scoring system for preoperative cardiovascular risk assessment in this high-risk population.

## Methods

### Study design and participants

Consecutive adult patients (aged ≥ 18 years) with established CAD undergoing elective non-cardiac surgery were included in this multicenter retrospective study. The study population was identified from two tertiary academic medical centers in Zhejiang Province, China: the First Affiliated Hospital of Zhejiang University School of Medicine (AHZU) (enrollment period: between January 1, 2013 and May 31, 2021) and the Fourth AHZU (between October 1, 2020 and October 31, 2024).

This study complied with the Declaration of Helsinki and was approved by the Institutional Review Boards (IRB) of both participating institutions (First AHZU: IIT20230114A; Fourth AHZU: K2024222). The retrospective design warranted waiver of informed consent. All data were anonymized before analysis.

All included procedures were elective non-cardiac surgeries, categorized based on established guideline criteria for perioperative cardiovascular evaluation [15]. CAD was diagnosed as previously described [16], defined by one or more of the following: a documented history of ≥ 50% coronary stenosis on angiography, prior myocardial infarction or coronary revascularization, or a clinical diagnosis consistent with standard guideline criteria[17]. We excluded emergency/day surgeries, repeat procedures during same hospitalization, and cases with incomplete data. Preoperative evaluation was conducted for all patients following established perioperative management guidelines.

### Data collection

We extracted data from both centers’ electronic medical records. Potential participants were identified *via* International Classification of Diseases, Tenth Revision (ICD-10) codes for CAD in surgical discharges, followed by manual screening and eligibility adjudication based on predefined criteria. The collected dataset encompassed demographics, preoperative evaluations, American Society of Anesthesiologists (ASA) classification, surgical and anesthetic details, perioperative cardiovascular complications, and other pertinent information. Data from January 1, 2013, to October 31, 2024, were analyzed in September 2025.

### Predictors

The AUB-HAS2 index assigns one point for each of the following six components: history of heart disease; symptoms of heart disease (angina or dyspnea); anemia (hemoglobin < 12 g/dL); age ≥ 75 years; emergency surgery; and vascular surgery [11]. In this context, “history of heart disease” was specifically defined as a documented history of coronary angioplasty, myocardial infarction, cardiac surgery, atrial fibrillation, heart failure, or moderate-to-severe valvular disease confirmed by echocardiography. It is important to note that although patients with CAD are commonly considered to have heart disease, not all CAD patients meet the specific criteria for this component as defined by the AUB-HAS2 index.

The RCRI is calculated by assigning one point for each of the following six risk factors: history of ischemic heart disease, congestive heart failure, or cerebrovascular disease; insulin-dependent diabetes mellitus; serum creatinine level > 2 mg/dL; and high-risk surgery (suprainguinal vascular, intraperitoneal, or intrathoracic procedures) [18].

### Outcome

The primary outcome measure was PCEs, a composite endpoint of all-cause death, myocardial infarction, or stroke. These events were assessed if they occurred intraoperatively or during hospitalization. Myocardial infarction and stroke were adjudicated using criteria from a prior study [16], in accordance with standard guidelines [19,20]. Of note, cardiac biomarkers were measured only when myocardial infarction was clinically suspected; routine systematic troponin monitoring was not performed. All potential events were independently adjudicated by reviewers blinded to clinical data.

### Statistical analysis

Data were managed with Microsoft Excel (Microsoft, Redmond, Washington) and analyzed using Statistical Package for Social Sciences (SPSS, version 23, IBM, Armonk, New York). Variable distribution was assessed *via* histograms and Q-Q plots. Continuous variables are presented as mean ± standard deviation (SD) if normally distributed, or as median with interquartile range (IQR) if non-normally distributed. Categorical variables are expressed as numbers and percentages (n, %). Group comparisons were made using ANOVA or the Kruskal–Wallis test for continuous variables, depending on their distribution, and the chi-squared test or Fisher’s exact test for categorical variables, as appropriate. Associations between predictors and outcomes were examined using univariate and multivariate logistic regression, with results reported as odds ratios (OR) and 95% confidence intervals (CI). Multivariable models were adjusted for potential confounders, including age, sex, body mass index, major comorbidities, ASA class, type of surgery, anesthesia method, and laboratory parameters. Trends were analyzed with the Mantel–Haenszel test. Model discrimination was evaluated using receiver operating characteristic (ROC) curves and the area under the curve (AUC), with differences in AUC compared *via* the DeLong test. Calibration was assessed visually using calibration plots, and clinical utility was evaluated with decision curve analysis (DCA). A two-tailed *p* < 0.05 was considered statistically significant.

## Results

### Baseline characteristics

**Figure 1.**
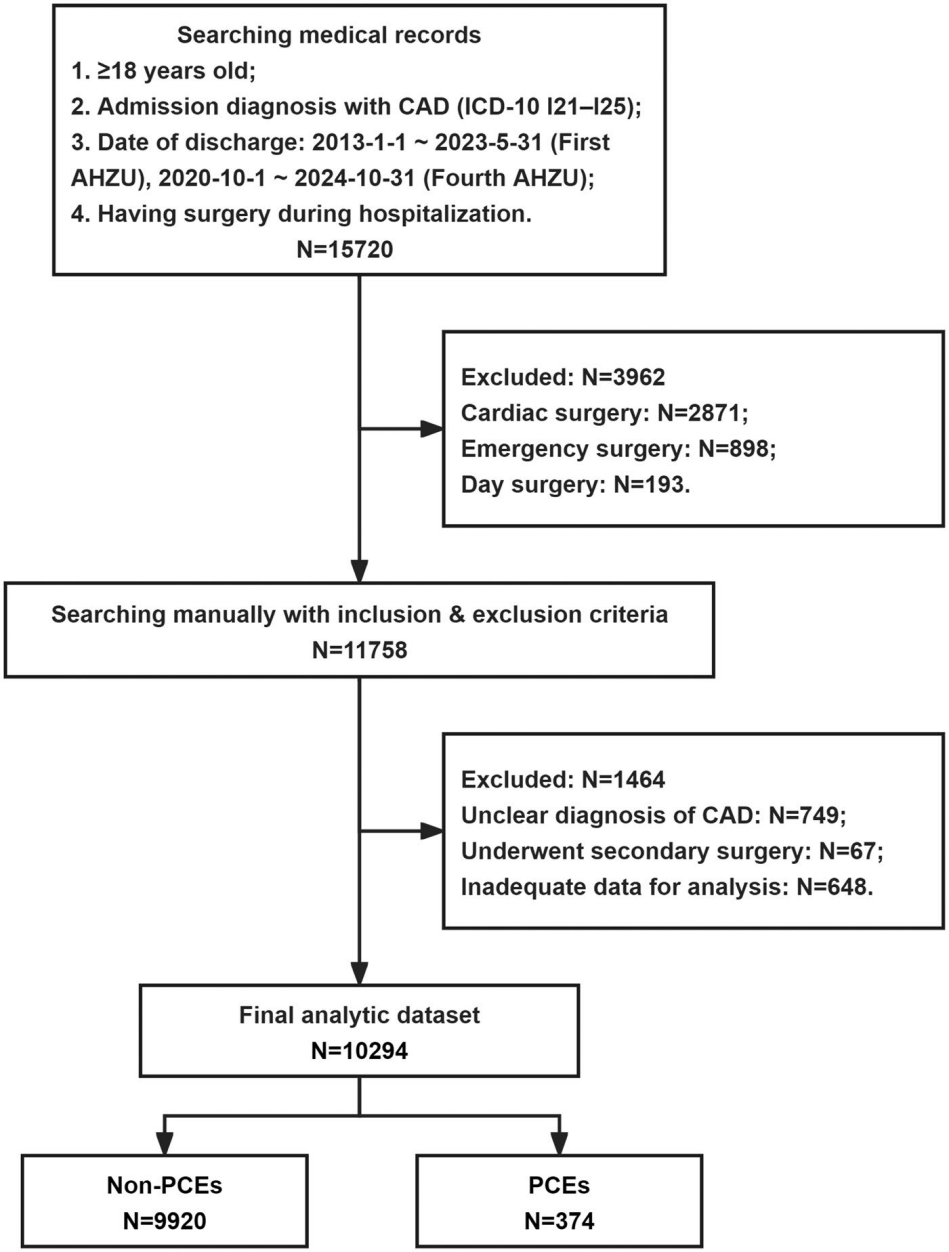
Flow chart of the patient enrollment and analysis. Abbreviations: CAD, coronary artery disease; ICD-10, International Classification of Diseases, Tenth Revision; AHZU, Affiliated Hospital of Zhejiang University School of Medicine; PCE, perioperative cardiovascular event.

This study included 10,294 patients with CAD who were aged 18 years or older and underwent non-cardiac surgery. The median age was 70 years (IQR, 63–76). Figure 1 depicts the patient enrollment and analysis flowchart. Baseline clinical characteristics and their associations with perioperative outcomes are detailed in Table 1.

**Table 1.** Baseline clinical characteristics and their association with perioperative outcomes.

Variables	Total (*n* = 10294)	Non-PCEs (*n* = 9920)	PCEs (*n* = 374)	P-value
Age (years)	70 [63, 76]	70 [63, 76]	73 [65, 79]	<0.001
Male	6806 (66.1)	6534 (65.9)	272 (72.7)	0.001
Body mass index (kg/m^2^)	23.73 [21.56, 25.86]	23.78 [21.63, 25.91]	22.49 [20.06, 24.78]	<0.001
Diabetes mellitus	2849 (27.7)	2709 (27.3)	140 (37.4)	<0.001
Hypertension	6558 (63.7)	6311 (63.6)	247 (66.0)	0.339
Stroke	964 (9.4)	907 (9.1)	57 (15.2)	<0.001
COPD	256 (2.5)	247 (2.5)	9 (2.4)	0.919
Dialysis	191 (1.9)	161 (1.6)	30 (8.0)	<0.001
Ischemic heart disease	4184 (40.6)	3962 (39.9)	222 (59.4)	<0.001
Myocardial infarction	2127 (20.7)	2031 (20.5)	96 (25.7)	0.015
Heart failure	552 (5.4)	496 (5.0)	56 (15.0)	<0.001
Atrial fibrillation	484 (4.7)	441 (4.4)	43 (11.5)	<0.001
Valvular heart disease	178 (1.7)	162 (1.6)	16 (4.3)	<0.001
Coronary angioplasty	2559 (24.9)	2474 (24.9)	85 (22.7)	0.331
CABG	180 (1.7)	175 (1.8)	5 (1.3)	0.536
Leukocyte (×10^9^/L)	6.1 [5.0, 7.5]	6.1 [5.0, 7.5]	7.0 [5.4, 10.2]	<0.001
Hemoglobin (g/L)	132 [117, 143]	132 [118, 144]	106 [86, 125]	<0.001
Platelet (×10^9^/L)	195 [158, 239]	195 [159, 239]	176 [132, 233]	<0.001
Creatinine (μmol/L)	76 [64, 92]	76 [64, 91]	89 [66, 137]	<0.001
ASA class				<0.001
II	4450 (43.2)	4388 (44.2)	62 (16.6)	
III	5760 (56.0)	5482 (55.3)	278 (73.3)	
IV	84 (0.8)	50 (0.5)	34 (9.1)	
Types of surgery				
General	2988 (29.0)	2849 (28.7)	139 (37.2)	<0.001
Abdominal	2240 (21.8)	2120 (21.4)	120 (32.1)	<0.001
Nonabdominal	748 (7.3)	729 (7.3)	19 (5.1)	0.097
Thoracic	1228 (11.9)	1199 (12.1)	29 (7.8)	0.011
Orthopedic	1420 (13.8)	1361 (13.7)	59 (15.8)	0.258
ENT	244 (2.4)	239 (2.4)	5 (1.3)	0.181
Neurological	435 (4.2)	402 (4.1)	33 (8.8)	<0.001
Gynecologic	224 (2.2)	218 (2.2)	6 (1.6)	0.440
Urologic	1830 (17.8)	1799 (18.1)	31 (8.3)	<0.001
Ophthalmology	689 (6.7)	687 (6.9)	2 (0.5)	<0.001
Vascular	1060 (10.3)	992 (10.0)	68 (18.2)	<0.001
Dental	176 (1.7)	174 (1.8)	2 (0.5)	0.074
General anesthesia	7675 (74.6)	7369 (74.3)	306 (81.8)	0.001
Duration of hospital stay (days)	8.8 [5.0, 14.0]	8.1 [5.0, 13.9]	17.8 [10.8, 27.2]	<0.001
RCRI	1 [0, 2]	1 [0, 2]	2 [1, 3]	<0.001
AUB-HAS2 index	1 [0, 2]	1 [0, 2]	2 [1, 3]	<0.001

Notes: Continuous variables are presented as median [IQR], and categorical variables as n (%).

Abbreviations: PCE, perioperative cardiovascular event; COPD, chronic obstructive pulmonary disease; CABG, coronary artery bypass graft; ASA, American Society of Anesthesiologists; ENT, ear, nose, and throat; RCRI, revised cardiac risk index; AUB, American University of Beirut.

The surgical procedures, conducted across two tertiary referral centers, encompassed a range of specialties. The most common types were general (29.0%), urologic (17.8%), orthopedic (13.8%), thoracic (11.9%), and vascular (10.3%) surgeries.

A total of 374 patients (3.6%) experienced PCEs. The group with PCEs, compared to the group without, was significantly older (median age: 73 vs. 70 years; *p* < 0.001), had a lower median body mass index (22.49 vs. 23.78 kg/m^2^; *p* < 0.001), and contained a higher percentage of male patients (72.7% vs. 65.9%; *p* = 0.001). Significant differences were also observed in comorbidities: the PCEs group had higher rates of diabetes mellitus (37.4% vs. 27.3%), stroke (15.2% vs. 9.1%), and dialysis (8.0% vs. 1.6%); as well as ischemic heart disease (59.4% vs. 39.9%), myocardial infarction (25.7% vs. 20.5%; *p* = 0.015), heart failure (15.0% vs. 5.0%), atrial fibrillation (11.5% vs. 4.4%), and valvular heart disease (4.3% vs. 1.6%) (all *p* < 0.001 unless specified). Furthermore, the distribution of ASA physical status was higher in the PCEs group, with 73.3% classified as ASA III (vs. 55.3%) and 9.1% as ASA IV (vs. 0.5%) (both *p* < 0.001).

A comparison of preoperative laboratory data showed significant differences. Patients with PCEs presented with higher leukocyte counts and creatinine levels, whereas their hemoglobin levels and platelet counts were lower.

Surgically, the PCEs group was characterized not only by a higher rate of general anesthesia (81.8% vs. 74.3%; *p* = 0.001) but also by a greater proportion of high-risk procedures, including general abdominal (32.1% vs. 21.4%), neurological (8.8% vs. 4.1%), and vascular surgery (18.2% vs. 10.0%) (all *p* < 0.001).

### Perioperative outcomes

Of the 374 patients with PCEs, myocardial infarction constituted the majority of complications (79.1%, *n* = 296). All-cause mortality occurred in 90 patients (24.1%), and stroke in 50 patients (13.4%). The detailed composition of all PCEs is provided in Table 2.

**Table 2.** Incidence of the perioperative outcomes stratified by the AUB-HAS2 index.

Outcomes	Participants (*N* = 10294)	AUB-HAS2 index					
		0 (*N* = 2853)	1 (*N* = 3950)	2 (*N* = 2261)	3 (*N* = 977)	> 3 (*N* = 253)	P-value
Death	90 (0.9)	2 (0.1)	14 (0.3)	26 (1.1)	30 (3.1)	18 (7.1)	<0.001
Myocardial infarction	296 (2.8)	5 (0.1)	67 (1.6)	94 (4.1)	79 (8.0)	51 (20.1)	<0.001
Stroke	50 (0.4)	2 (0.1)	21 (0.5)	14 (0.6)	9 (0.9)	4 (1.5)	<0.001
Total	374 (3.6)	9 (0.3)	92 (2.3)	119 (5.2)	99 (10.1)	55 (21.7)	<0.001

Notes: Results are presented as n (%).

Abbreviations: AUB, American University of Beirut.

### Association between predictors and perioperative outcomes

Results from the univariate and multivariate analyses are summarized in Table 3. While every component of the AUB-HAS2 index demonstrated independent predictive value for PCEs (all *p* < 0.05), the high-risk surgery component of the RCRI failed to retain a significant association following adjustment for confounding variables (OR = 0.984; 95% CI: 0.774–1.250; *p* = 0.893).

**Table 3.** Univariate and multivariate analyses of AUB-HAS2 index and RCRI associations with perioperative outcomes.

Variables	Events	Univariate regression		Multivariate regression	
	% (n/N)	OR (95% CI)	P-value	OR (95% CI)	P-value
AUB-HAS2 index components					
History of heart disease					
No	3.1 (207/6647)	Reference		Reference	
Yes	4.6 (167/3647)	1.493 (1.213, 1.838)	<0.001	1.300 (1.036, 1.631)	0.024
Symptoms of heart disease (angina or dyspnea)					
No	2.0 (170/8578)	Reference		Reference	
Yes	11.9 (204/1716)	6.673 (5.405, 8.239)	<0.001	4.436 (3.529, 5.576)	<0.001
Age ≥ 75 years					
No	3.0 (213/7176)	Reference		Reference	
Yes	5.2 (161/3118)	1.780 (1.444, 2.194)	<0.001	1.556 (1.100, 2.203)	0.013
Anemia (hemoglobin < 12 g/dL)					
No	1.6 (119/7395)	Reference		Reference	
Yes	8.8 (255/2899)	5.897 (4.722, 7.363)	<0.001	2.659 (2.028, 3.487)	<0.001
Vascular surgery					
No	3.3 (306/9234)	Reference		Reference	
Yes	6.4 (68/1060)	2.000 (1.525, 2.622)	<0.001	1.520 (1.101, 2.097)	0.011
RCRI components					
History of ischemic heart disease					
No	2.5 (152/6110)	Reference		Reference	
Yes	5.3 (222/4184)	2.196 (1.780, 2.710)	<0.001	1.767 (1.408, 2.218)	<0.001
History of congestive heart failure					
No	3.3 (318/9742)	Reference		Reference	
Yes	10.1 (56/552)	3.346 (2.484, 4.508)	<0.001	1.484 (1.058, 2.081)	0.022
History of cerebrovascular disease					
No	3.4 (317/9330)	Reference		Reference	
Yes	5.9 (57/964)	1.787 (1.337, 2.388)	0.001	1.566 (1.135, 2.160)	0.006
Insulin-dependent diabetes mellitus					
No	3.1 (261/8507)	Reference		Reference	
Yes	6.3 (113/1787)	2.133 (1.700, 2.676)	<0.001	1.531 (1.192, 1.966)	0.001
Creatinine > 2 mg/dL					
No	3.1 (302/9830)	Reference		Reference	
Yes	15.5 (72/464)	5.795 (4.396, 7.638)	<0.001	2.089 (1.483, 2.943)	<0.001
High-risk surgery					
No	3.1 (201/6447)	Reference		Reference	
Yes	4.5 (173/3847)	1.463 (1.189, 1.800)	<0.001	0.984 (0.774, 1.250)	0.893

Abbreviations: AUB, American University of Beirut; RCRI, revised cardiac risk index; OR, odds ratio; CI, confidence interval.

### Model performance

The comparison of PCEs among the AUB-HAS2 index groups in the study cohort is shown in Table 2. The incidence of the primary outcome increased steadily with the increase in AUB-HAS2 index (0.3% for AUB-HAS2 index of 0, 2.3% for AUB-HAS2 index of 1, 5.2% for AUB-HAS2 index of 2, 10.1% for AUB-HAS2 index of 3, and 21.7% for AUB-HAS2 index > 3; p value for trend < 0.001).

**Figure 2.**
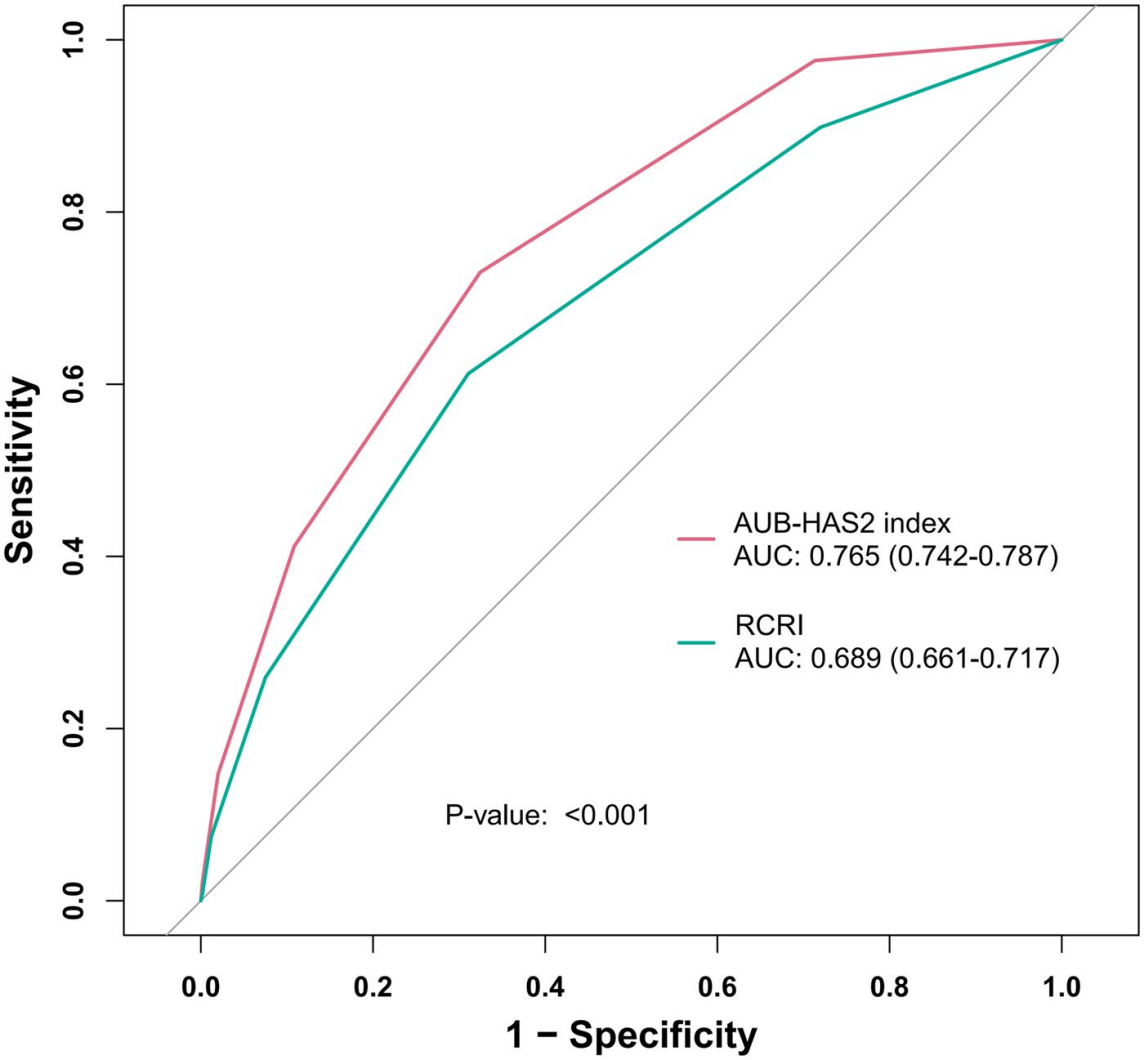
Receiver operating characteristic curves for AUB-HAS2 index and RCRI. Abbreviations: AUB, American University of Beirut; RCRI, revised cardiac risk index; AUC, area under the receiver operating characteristic curve.

**Figure 3.**
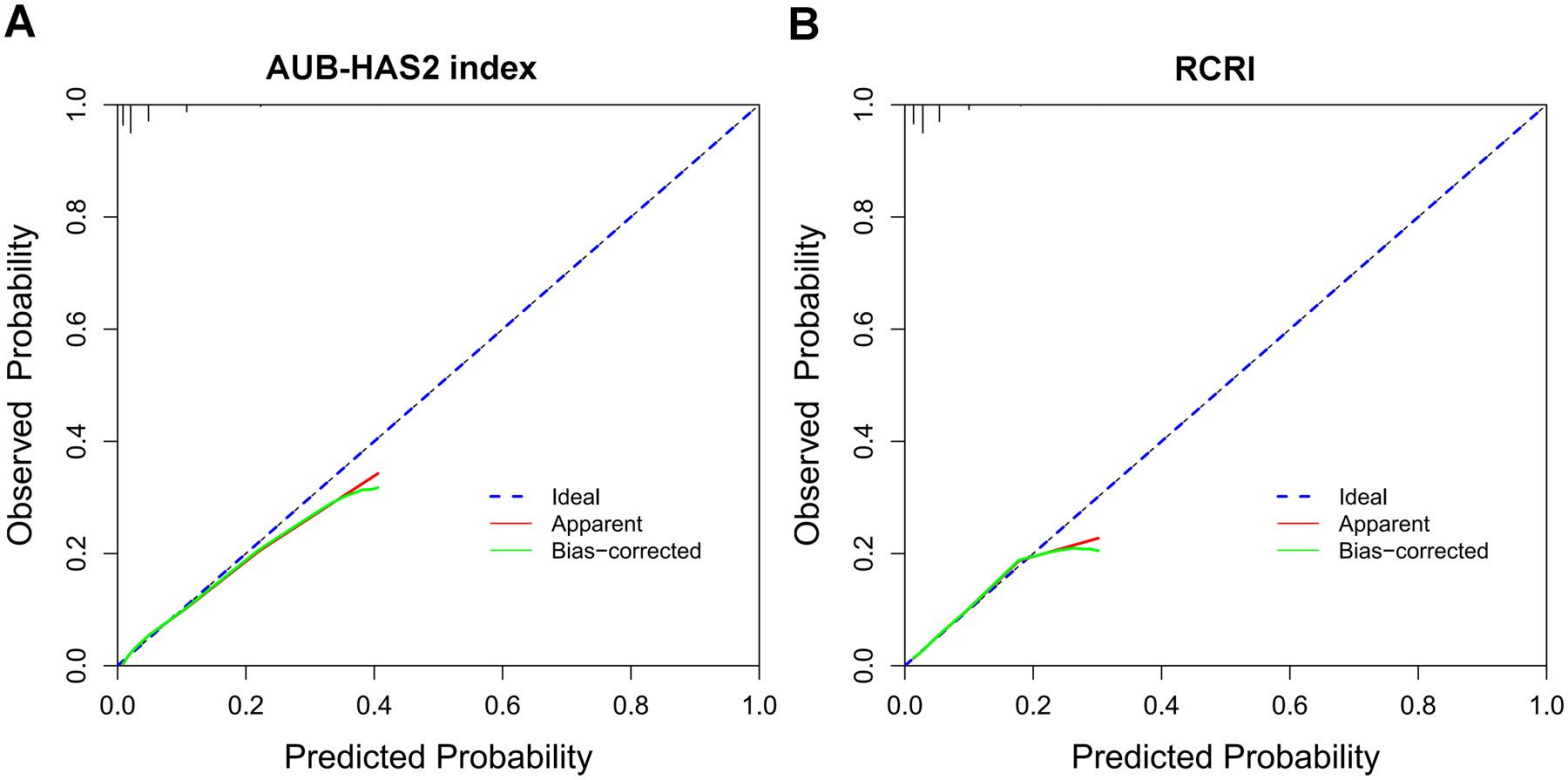
Calibration curves of AUB-HAS2 index (A) and RCRI (B). Abbreviations: AUB, American University of Beirut; RCRI, revised cardiac risk index.

**Figure 4.**
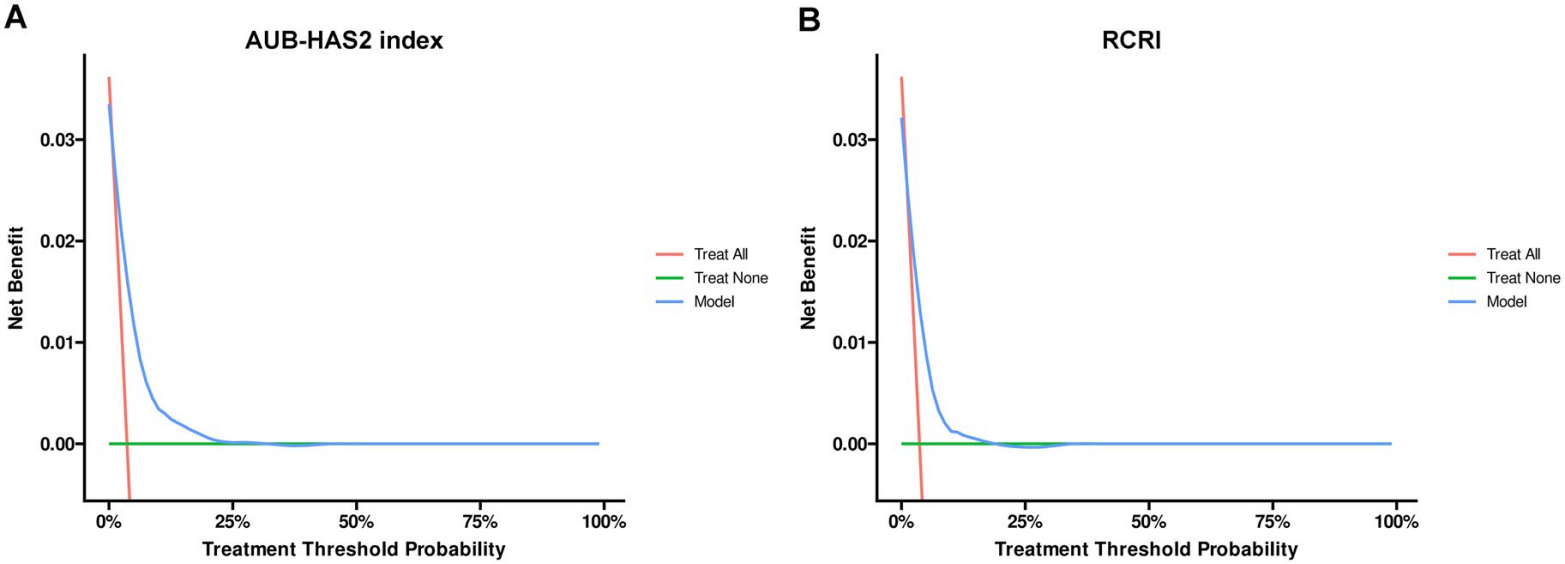
Decision curve analysis of AUB-HAS2 index (A) and RCRI (B). Abbreviations: AUB, American University of Beirut; RCRI, revised cardiac risk index.

The discriminatory power of the AUB-HAS2 index and the RCRI was compared using ROC curves (Figure 2). The AUB-HAS2 index demonstrated a significantly higher AUC than the RCRI (0.765 vs. 0.689; *p* < 0.001). Both models showed good calibration by visual inspection of calibration plots (Figure 3), with the AUB-HAS2 index appearing better aligned. Decision curve analysis indicated that both models provided clinical net benefit across a wide range of threshold probabilities (Figure 4); however, the AUB-HAS2 index offered improved net benefit over a broader probability range.

### Subgroup analysis

The AUC values of the AUB-HAS2 index and the RCRI across population subgroups are compared in Table 4. Consistently, the AUB-HAS2 index demonstrated improved discriminatory ability to the RCRI in all subgroups. However, this improvement did not reach statistical significance in three specific cohorts: patients with diabetes mellitus, those who received non-general anesthesia, and those undergoing certain surgical procedures (orthopedic, neurological, gynecologic, ophthalmologic, dental, or ear, nose, and throat surgeries).

**Table 4.** AUC comparison between AUB-HAS2 index and RCRI in population subgroups.

Subgroups	AUB-HAS2 index	RCRI	P-value
	AUC (95%CI)	AUC (95%CI)	
Age (years)			
≥ 65	0.752 (0.724–0.780)	0.679 (0.647–0.712)	<0.001
< 65	0.806 (0.763–0.849)	0.715 (0.660–0.770)	<0.001
Sex			
Male	0.761 (0.734–0.788)	0.706 (0.675–0.738)	<0.001
Female	0.774 (0.730–0.818)	0.636 (0.580–0.692)	<0.001
Hypertension			
No	0.776 (0.739–0.814)	0.673 (0.627–0.718)	<0.001
Yes	0.758 (0.729–0.787)	0.698 (0.663–0.733)	0.001
Diabetes mellitus			
No	0.778 (0.749–0.807)	0.671 (0.635–0.707)	<0.001
Yes	0.732 (0.694–0.770)	0.697 (0.653–0.742)	0.15
Types of surgery			
General	0.766 (0.729–0.803)	0.657 (0.611–0.702)	<0.001
Abdominal	0.825 (0.736–0.915)	0.705 (0.605–0.805)	0.004
Nonabdominal	0.748 (0.707–0.789)	0.629 (0.575–0.682)	<0.001
Thoracic	0.758 (0.684–0.833)	0.606 (0.491–0.721)	0.008
Orthopedic	0.733 (0.672–0.793)	0.713 (0.639–0.788)	0.594
ENT	0.770 (0.642–0.898)	0.655 (0.434–0.876)	0.32
Neurological	0.693 (0.608–0.777)	0.647 (0.542–0.753)	0.47
Gynecologic	0.781 (0.618–0.944)	0.739 (0.499–0.979)	0.713
Urologic	0.780 (0.704–0.857)	0.644 (0.526–0.761)	0.019
Ophthalmology	0.935 (0.857–1.000)	0.905 (0.764–1.000)	0.499
Vascular	0.751 (0.690–0.813)	0.667 (0.600–0.734)	0.021
Dental	0.904 (0.794–1.000)	0.632 (0.142–1.000)	0.313
General anesthesia			
No	0.754 (0.700–0.808)	0.728 (0.672–0.783)	0.392
Yes	0.774 (0.749–0.799)	0.676 (0.643–0.708)	<0.001

Abbreviations: AUC, area under the receiver operating characteristic curve; AUB, American University of Beirut; RCRI, revised cardiac risk index; CI, confidence interval; ENT, ear, nose, and throat.

## Discussion

This multicenter retrospective cohort study validated the AUB-HAS2 index in CAD patients undergoing non-cardiac surgery. The index demonstrated excellent risk stratification performance, effectively categorizing patients into low-, intermediate-, and high-risk groups. Compared with the RCRI, the AUB-HAS2 index showed improved discriminative capacity, better calibration, and enhanced clinical utility across decision thresholds.

To our knowledge, this study provides the first validation of the AUB-HAS2 index specifically in CAD patients undergoing non-cardiac surgery. Given that CAD patients represent a particularly high-risk surgical population, accurate preoperative risk stratification is critical for implementing appropriate risk mitigation strategies and improving perioperative outcomes [21]. This study therefore addresses an important clinical need while contributing to the ongoing validation of this novel risk assessment tool.

Our analysis demonstrated improved discriminative performance of the AUB-HAS2 index compared to the RCRI in CAD patients, which can be attributed to four key methodological advantages. First, the AUB-HAS2 index expands upon the RCRI’s cardiac history assessment by incorporating active cardiac conditions such as angina and dyspnea, both of which demonstrated independent associations with PCEs (Table 3). Second, it provides more contemporary surgical risk stratification by appropriately classifying vascular surgery as high-risk [22], whereas the RCRI’s classification of intrathoracic procedures as high-risk may be outdated, given the lower complication rates associated with modern minimally invasive approaches [23]. Third, unlike the RCRI, the AUB-HAS2 index derivation cohort included low-risk surgical patients, thereby enhancing its clinical applicability. Finally, its composite endpoint includes non-cardiovascular mortality and stroke, outcomes not captured by the RCRI, thus offering a more comprehensive assessment of perioperative risk.

This study confirms that CAD patients with an AUB-HAS2 score of 0 exhibit minimal perioperative cardiovascular risk (0.3%, < 1%). This finding carries significant clinical implications, as this low-risk subgroup represents a substantial proportion (27.7%) of the CAD population. The simplicity of the AUB-HAS2 index facilitates rapid preoperative triage, potentially obviating the need for additional cardiovascular testing or routine postoperative monitoring in these low-risk patients. This approach aligns with current guideline recommendations to avoid low-value preoperative interventions in low-risk populations [9].

Conversely, the index reliably identifies high-risk patients (score > 3) with substantially elevated perioperative cardiovascular risk (21.7%). For these individuals, the AUB-HAS2 index supports a more intensive management approach, including comprehensive preoperative cardiovascular assessment, optimization of guideline-directed medical therapy, and enhanced postoperative surveillance. By enabling early differentiation of risk profiles, the AUB-HAS2 index facilitates individualized perioperative care and promotes efficient resource allocation. Integration of this simple, validated tool into routine preoperative evaluation may therefore complement existing perioperative guidelines and improve clinical outcomes across the full spectrum of surgical risk [12].

While our study benefits from a multicenter design with substantial sample size, several limitations should be noted. First, the retrospective study design inherently carries risks of selection and information bias. Second, although this study was conducted across two tertiary medical centers with a large cohort, its single-country design may limit generalizability. Variations in patient demographics and perioperative practices across different healthcare systems could introduce geographic or systemic bias. Accordingly, future multicenter studies involving more diverse populations spanning different geographic regions and healthcare systems are needed to validate our findings and enhance external validity. Third, the reported PCE incidence might underestimate the true event rates, as clinically silent myocardial infarctions—which can only be identified through routine biomarker screening—were not included in the endpoint assessment [24]. This exclusion likely results in the misclassification of certain silent infarctions as non-PCEs, thereby attenuating the apparent performance of the model in terms of AUC, calibration, and DCA. Fourth, while the AUB-HAS2 index demonstrated improved discriminatory power relative to the RCRI across all subgroups, this improvement did not reach statistical significance in three specific cohorts: patients with diabetes mellitus, those receiving non-general anesthesia, and those undergoing certain surgical procedures (orthopedic, neurological, gynecologic, ophthalmologic, dental, or ear, nose, and throat surgeries). Notably, the true effect in these subgroups remains underexplored, and future studies specifically targeting these populations are warranted to confirm the consistency of the observed improvement.

## Conclusions

This study extends the validation of the AUB-HAS2 index in CAD population, demonstrating its improved discriminatory power compared with the widely used RCRI. These findings support the clinical adoption of AUB-HAS2 index for preoperative cardiovascular risk stratification in this high-risk population. Nevertheless, further validation through multicenter prospective studies with longer follow-up period is warranted to confirm these results.

## Supplementary Material

manuscript revision 2 clean copy.docx

## Data Availability

The datasets used and/or analysed during the current study are available from the corresponding author on reasonable request.

## References

[1] Smilowitz NR, Gupta N, Guo Y, et al. Trends in cardiovascular risk factor and disease prevalence in patients undergoing non-cardiac surgery. Heart. 2018;104(14):1180–1186. doi: 10.1136/heartjnl-2017-312391. 29305561 PMC6102124

[2] Weiser TG, Haynes AB, Molina G, et al. Size and distribution of the global volume of surgery in 2012. Bull World Health Organ. 2016;94(3):201–209F. doi: 10.2471/BLT.15.159293m. 26966331 PMC4773932

[3] Xu L, Yu C, Jiang J, et al. Major adverse cardiac events in elderly patients with coronary artery disease undergoing noncardiac surgery: a multicenter prospective study in China. Arch Gerontol Geriatr. 2015;61(3):503–509. doi: 10.1016/j.archger.2015.07.006. 26272285

[4] Holcomb CN, Graham LA, Richman JS, et al. The incremental risk of coronary stents on postoperative adverse events: a matched cohort study. Ann Surg. 2016;263(5):924–930. doi: 10.1097/SLA.0000000000001246. 25894416

[5] Smilowitz NR, Gupta N, Ramakrishna H, et al. Perioperative major adverse cardiovascular and cerebrovascular events associated with noncardiac surgery. JAMA Cardiol. 2017;2(2):181–187. doi: 10.1001/jamacardio.2016.4792. 28030663 PMC5563847

[6] Handke J, Scholz AS, Dehne S, et al. Presepsin for pre-operative prediction of major adverse cardiovascular events in coronary heart disease patients undergoing noncardiac surgery: post hoc analysis of the Leukocytes and Cardiovascular Peri-operative Events-2 (LeukoCAPE-2) Study. Eur J Anaesthesiol. 2020;37(10):908–919. doi: 10.1097/EJA.0000000000001243. 32516228

[7] Siddiqui E, Banco D, Berger JS, et al. Frailty assessment and perioperative major adverse cardiovascular events after noncardiac surgery. Am J Med. 2023;136(4):372–379.e5. doi: 10.1016/j.amjmed.2022.12.033. 36657557 PMC10038881

[8] Halvorsen S, Mehilli J, Cassese S, et al. 2022 ESC Guidelines on cardiovascular assessment and management of patients undergoing non-cardiac surgery. Eur Heart J. 2022;43(39):3826–3924. doi: 10.1093/eurheartj/ehac270. 36017553

[9] Thompson A, Fleischmann KE, Smilowitz NR, et al. 2024 AHA/ACC/ACS/ASNC/HRS/SCA/SCCT/SCMR/SVM guideline for perioperative cardiovascular management for noncardiac surgery: a report of the American college of cardiology/American heart association joint committee on clinical practice guidelines. Circulation. 2024;150(19):e351–e442. doi: 10.1161/CIR.0000000000001285. 39316661

[10] Che L, Xu L, Huang Y, et al. Clinical utility of the revised cardiac risk index in older Chinese patients with known coronary artery disease. Clin Interv Aging. 2018;13:35–41. doi: 10.2147/CIA.S144832. 29317808 PMC5743178

[11] Dakik HA, Chehab O, Eldirani M, et al. A new index for pre-operative cardiovascular evaluation. J Am Coll Cardiol. 2019;73(24):3067–3078. doi: 10.1016/j.jacc.2019.04.023. 31221255

[12] Dakik HA, Sbaity E, Msheik A, et al. AUB-HAS2 cardiovascular risk index: performance in surgical subpopulations and comparison to the revised cardiac risk index. J Am Heart Assoc. 2020;9(10):e016228. doi: 10.1161/JAHA.119.016228. 32390481 PMC7660845

[13] Msheik A, Kaspar C, Mailhac A, et al. Performance of the AUB-HAS2 cardiovascular risk index in vascular surgery patients. Vasc Med. 2021;26(5):535–541. doi: 10.1177/1358863X21996806. 33813967

[14] Dakik HA, Eldirani M, Kaspar C, et al. Prospective validation of the AUB-HAS2 cardiovascular risk index. Eur Heart J Qual Care Clin Outcomes. 2022;8(1):96–97. doi: 10.1093/ehjqcco/qcaa077. 33017006

[15] Fleisher LA, Fleischmann KE, Auerbach AD, et al. 2014 ACC/AHA guideline on perioperative cardiovascular evaluation and management of patients undergoing noncardiac surgery: executive summary: a report of the American College of Cardiology/American Heart Association Task Force on Practice Guidelines. Circulation. 2014;130(24):2215–2245. doi: 10.1161/CIR.0000000000000105. 25085962

[16] Li X, Wang C, Jiang H, et al. Geriatric nutritional risk index predicts perioperative cardiovascular events in older patients with coronary artery disease undergoing non-cardiac surgery: a multicenter retrospective cohort study. Front Nutr. 2025;12:1652742. doi: 10.3389/fnut.2025.1652742. 41141258 PMC12549302

[17] Fihn SD, Gardin JM, Abrams J, et al. 2012 ACCF/AHA/ACP/AATS/PCNA/SCAI/STS guideline for the diagnosis and management of patients with stable ischemic heart disease: executive summary: a report of the American College of Cardiology Foundation/American Heart Association task force on practice guidelines, and the American College of Physicians, American Association for Thoracic Surgery, Preventive Cardiovascular Nurses Association, Society for Cardiovascular Angiography and Interventions, and Society of Thoracic Surgeons. Circulation. 2012;126(25):3097–3137. doi: 10.1161/CIR.0b013e3182776f83. 23166210

[18] Lee TH, Marcantonio ER, Mangione CM, et al. Derivation and prospective validation of a simple index for prediction of cardiac risk of major noncardiac surgery. Circulation. 1999;100(10):1043–1049. doi: 10.1161/01.cir.100.10.1043. 10477528

[19] Thygesen K, Alpert JS, Jaffe AS, et al. Third universal definition of myocardial infarction. J Am Coll Cardiol. 2012;60(16):1581–1598. doi: 10.1016/j.jacc.2012.08.001. 22958960

[20] Sacco RL, Kasner SE, Broderick JP, et al. An updated definition of stroke for the 21st century: a statement for healthcare professionals from the American Heart Association/American Stroke Association. Stroke. 2013;44(7):2064–2089. doi: 10.1161/STR.0b013e318296aeca. 23652265 PMC11078537

[21] Cao D, Chandiramani R, Capodanno D, et al. Non-cardiac surgery in patients with coronary artery disease: risk evaluation and periprocedural management. Nat Rev Cardiol. 2021;18(1):37–57. doi: 10.1038/s41569-020-0410-z. 32759962

[22] Lee C, Columbo JA, Stone DH, et al. Preoperative evaluation and perioperative management of patients undergoing major vascular surgery. Vasc Med. 2022;27(5):496–512. doi: 10.1177/1358863X221122552. 36214163 PMC9551317

[23] Lin Y, Vervoort D, Thapa B, et al. Minimally invasive thoracic surgery for low- and middle-income countries. Thorac Surg Clin. 2022;32(3):405–412. doi: 10.1016/j.thorsurg.2022.04.003. 35961748

[24] Botto F, Alonso-Coello P, Chan MT, et al. Myocardial injury after noncardiac surgery: a large, international, prospective cohort study establishing diagnostic criteria, characteristics, predictors, and 30-day outcomes. Anesthesiology. 2014;120(3):564–578. doi: 10.1097/ALN.0000000000000113. 24534856

